# An optimized protocol to assess SUMOylation in the plant *Capsella rubella* using two-component DEX-inducible transformants

**DOI:** 10.1016/j.xpro.2022.101197

**Published:** 2022-02-26

**Authors:** Yang Dong, Zhi-Cheng Hu, Lars Østergaard

**Affiliations:** 1 State Key Laboratory of Systematic and Evolutionary Botany, Institute of Botany, Chinese Academy of Sciences, 20 Nanxincun, Xiangshan, Beijing 100093, China; 2 University of Chinese Academy of Sciences, Beijing 100049, China; 3 Crop Genetics Department, John Innes Centre, NR4 7UH Norwich, UK

**Keywords:** Model Organisms, Plant sciences, Molecular Biology, Protein Biochemistry

## Abstract

Here, we present an efficient protocol to test the SUMOylation of a target protein in the plant *Capsella rubella* based on overexpression of dexamethasone (DEX)-inducible tagged proteins. We describe the construction of two-component, FLAG-tagged DEX-inducible plasmids. We then detail the transformation of *Capsella,* followed by DEX treatment and SUMOylation assays. This protocol can be widely applied to proteins with expression restricted to specific cells and tissues using native promoters as well as proteins whose overexpression leads to embryo lethality.

For complete details on the use and execution of this profile, please refer to Dong et al. (2020).

## Highlights

• An easy-to-use pipeline for constructing FLAG-tagged DEX-inducible plasmids
• An efficient transformation protocol for *Capsella rubella*
• An optimized protocol to test SUMOylation of a protein of interest in plants

## Graphical abstract

**Figure:**
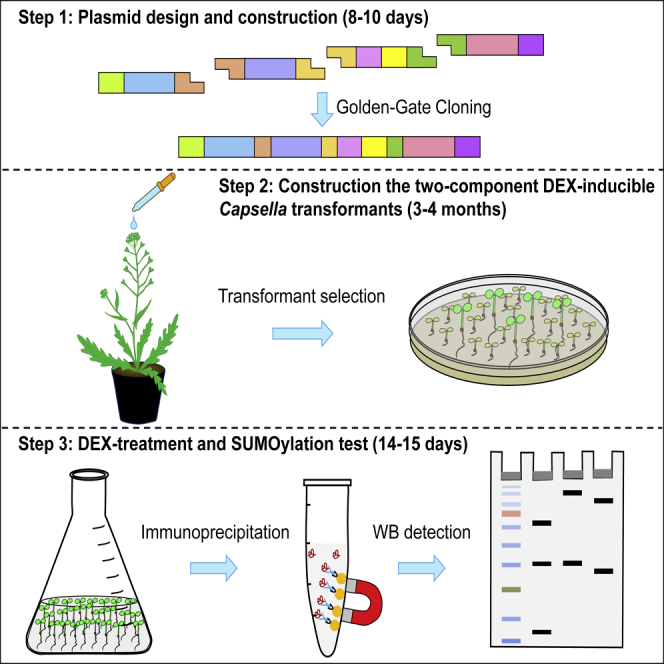

## Before you begin

Conjugation of small ubiquitin-related modifier (SUMO) to the proteins provides the plants with a fast and dynamic regulatory mechanism to cope with environmental fluctuations and intrinsic developmental clues (Mukhopadhyay and Dasso, 2007). Similar to ubiquitin, SUMO becomes covalently attached to lysyl ε-amino groups in the target proteins by the action of a dedicated enzymatic cascade (Miura and Hasegawa, 2010). Upon conjugation, SUMOylation usher in profound impact on the target protein, including the way they interact with other proteins and nucleic acids, stability and subcellular localization (Mukhopadhyay and Dasso, 2007). Interestingly, SUMOylation is a highly dynamic and reversible process as the SUMOs can be trimmed off by specific cysteine proteases in developmental-context dependent manner (Miura and Hasegawa, 2010). All these features make detection of SUMOylation a challenge in analyzing the biochemical property of a protein of interest.

Recently, we found that changes in regulatory region(s) of the *CrIND* gene compared to *Arabidopsis* lead to expressional expansion of *CrIND* from the valve margin to the valves in the cap, leading to the development of a heart-shaped fruit (Dong et al., 2019). In the valve tips, *CrIND* directly activates the expression of *CrTAA1* and *CrYUC9*, which in turn results in auxin maxima that affect cell anisotropic growth behavior in the valves and establish the heart (Dong et al., 2019). The CrIND protein function is fine-tuned at the post-translational level by SUMOylation as mutations in HTB, which encodes SUMO proteases, destabilize CrIND by SUMOylation (Dong et al., 2020). *CrIND* encodes a b-HLH transcription factor and exhibits a tissue specific expression pattern (Liljegren et al., 2004; Dong et al., 2019). In addition, over-expression of *CrIND* results in dramatic changes in the organ polarity and thus brings about plant lethality at the seedling stage (Moubayidin and Østergaard, 2014). Therefore, detection of CrIND SUMOylation is very challenging when expressing *CrIND* under the control of native promoter or constitutive promoter. Here, we describe a detailed SUMOylation test protocol as applied to *Capsella* seedlings transformed with a two-component DEX-inducible system to express FLAG-tagged protein, using CrIND as an example. It should be noted that this protocol is set up and optimized for *Capsella*, but could also be widely used to test the role of SUMOylation in diverse proteins from transformable non-model organisms.

### Experimental considerations

Before starting this protocol, please ensure that the instruments and equipment required for protein extraction and immunoprecipitation (IP) at 4°C are available as these steps are crucial for the detecting protein SUMOylation.

### Preparation of reagents and equipment

Refer to “key resources table” and “materials and equipment” for the lists of reagents and equipment.

### Construction of two-component DEX-inducible plasmids (*pLhGR*)

**Timing: ∼8–10 days**

The construction protocol is based on Golden-Gate cloning methods (Weber et al., 2011). Before doing the following experiment, please ensure that the coding sequence of the interested gene DO NOT possess any *Bsa*I and *Bbs*I (*Bpi*I) restriction site. If the sequence has any one of these two restriction sites, please domesticate the sites via PCRs.

1. Design the primers with 5′ sequence added the *Bpi*I sites compatible with the L0 acceptor for target sequence amplification (See Table S1 for an example on how to design the primer for the L0 reaction).
2. Amplify the target sequence (Coding Sequence) from cDNAs using high-fidelity enzyme.

***Note:*** To avoid sequence errors in the PCR products, we recommend using high-fidelity DNA polymerase.

3. Purify the PCR product from the 1.5% agarose gel.

**CRITICAL:** Always purify the PCR product from the gel, as it is key to the successful L0 reaction in the following step.

**Figure 1:**
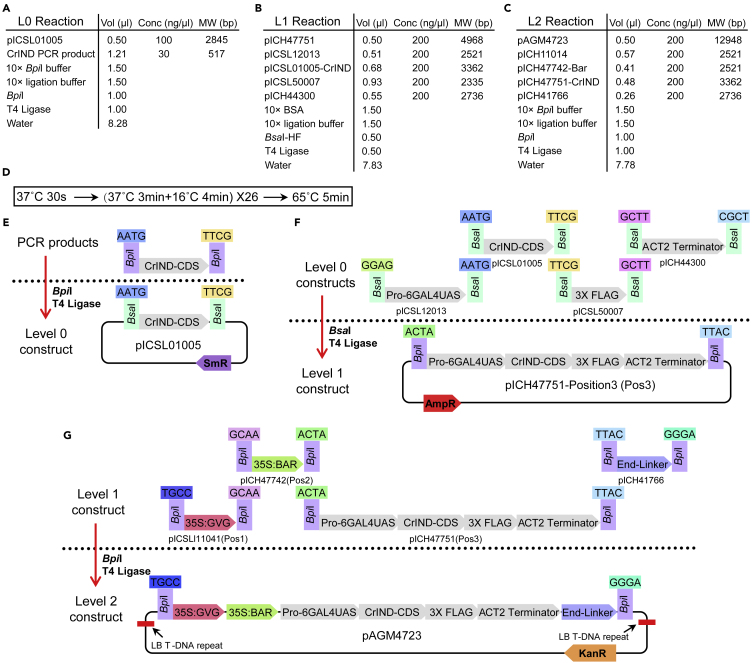
Details of the two-component DEX-inducible plasmid construction using Golden-Gate cloning method (A–C) Golden-Gate reaction set-up details of level 0 (L0, A), L1 (B) and L2 (C). (D) The Golden-Gate reaction program for L0-L2cloning. (E) A detailed overview of the organization and insertion of the target gene CDS (*CrIND*) in the L0 constructs (pICSL01005) through L0 reaction. (F) Assemble of different L0 modules to construct the *p6GAL4UAS*:CrIND:3×FLAG plasmid on the pICH47751 L1 plasmid. (G) Assemble of different L1 modules to construct the two-component DEX-inducible *pLhGR*>>CrIND:3×FLAG plasmid on the pAGA4723 L2 destination binary vector.

4. Golden-Gate L0 reaction using *Bpi*I and T4 ligase (for reaction and program set up, see Figures 1A and 1D, and Table S2).

**CRITICAL:** Always use the 15 μL reaction volumes and reaction program detailed in Figure 1 and Table S2.

5. Transform the reaction product into DH5-alpha competent *Escherichia coli* cells in a 1:10 ratio. Put 10–20 μL *E. coli* cells on LB plate containing 50 mg/L spectinomycin, 25 mg/L Isopropyl β-D-1-thiogalactopyranoside (IPTG) and 40 mg/L 5-Bromo-4-chloro-3-indolyl β-D-galactopyranoside (X-gal), grow the bacterial at 37°C for 14 h.

***Note:*** There is no special requirement for the *E. coli* competent cell, we recommend the DH5-alpha cell strain because of the high efficiency. Higher ratio between the plasmid and *E. coli* competent cells will result in lower transformation rate.

6. Pick 2–4 white colonies (against blue ones) and extract the plasmids by Midi-Prep and verify the insertion by sequencing.

**CRITICAL:** Always prepare the plasmid by Midi-Prep as it is critical for multiple DNA assemblies in the following step.

7. Golden-Gate L1 reaction using *Bsa*I and T4 ligase (for reaction and program set up, see Figures 1B and 1D, and Table S2).
8. Transform the reaction product into DH5-alpha competent *Escherichia coli* cells in a 1:10 ratio and put 10–20 μL on LB plate containing 100 mg/L Carbenicillin, 25 mg/L IPTG and 40 mg/L X-gal, grow the bacterial at 37°C for 14 h.

***Note:*** Higher ratio between the plasmid and *E. coli* competent cells will result in lower transformation rate.

9. Pick 2–4 white colonies (against blue ones) and extract the plasmids by Midi-Prep and verify the insertion by sequencing.
10. Golden-Gate L2 reaction using *Bpi*I and T4 ligase (for reaction and program set up, see Figures 1C and 1D, and Table S2).
11. Transform the reaction product into DH5-alpha competent *Escherichia coli* cells in a 1:10 ratio and put 20–30 μL on LB plate containing 50 mg/L Kanamycin, grow the bacterial at 37°C for 14 h.

***Note:*** Higher ratio between the plasmid and *E. coli* competent cells will result in lower transformation rate.

12. Pick 1–2 white colonies (against orange ones) and extract the plasmids and verify the insertion by sequencing.

### Prepare the two-component DEX-inducible *Capsella* transformants

**Timing: ∼3–4 months**

In the following section, we describe the detailed construction process of the two component DEX-inducible system (*pLhGR>>*) for the CrIND protein in the *Capsella htb-1* mutant, which harbors a loss-of-function mutation in the SUMO protease (Dong et al., 2020). For the mutant CrIND protein, full length *CrIND* coding sequence with lysine at position 124 (K124) mutated to arginine (R) was amplified by recombination PCR. We used the above mentioned Golden-gate cloning protocol to generate the *pLhGR*>>CrIND^K124R^:3×FLAG plasmids. The transformation protocol of *Capsella* followed the floral dipping method described in Dong et al. (2019).

**Figure 2:**
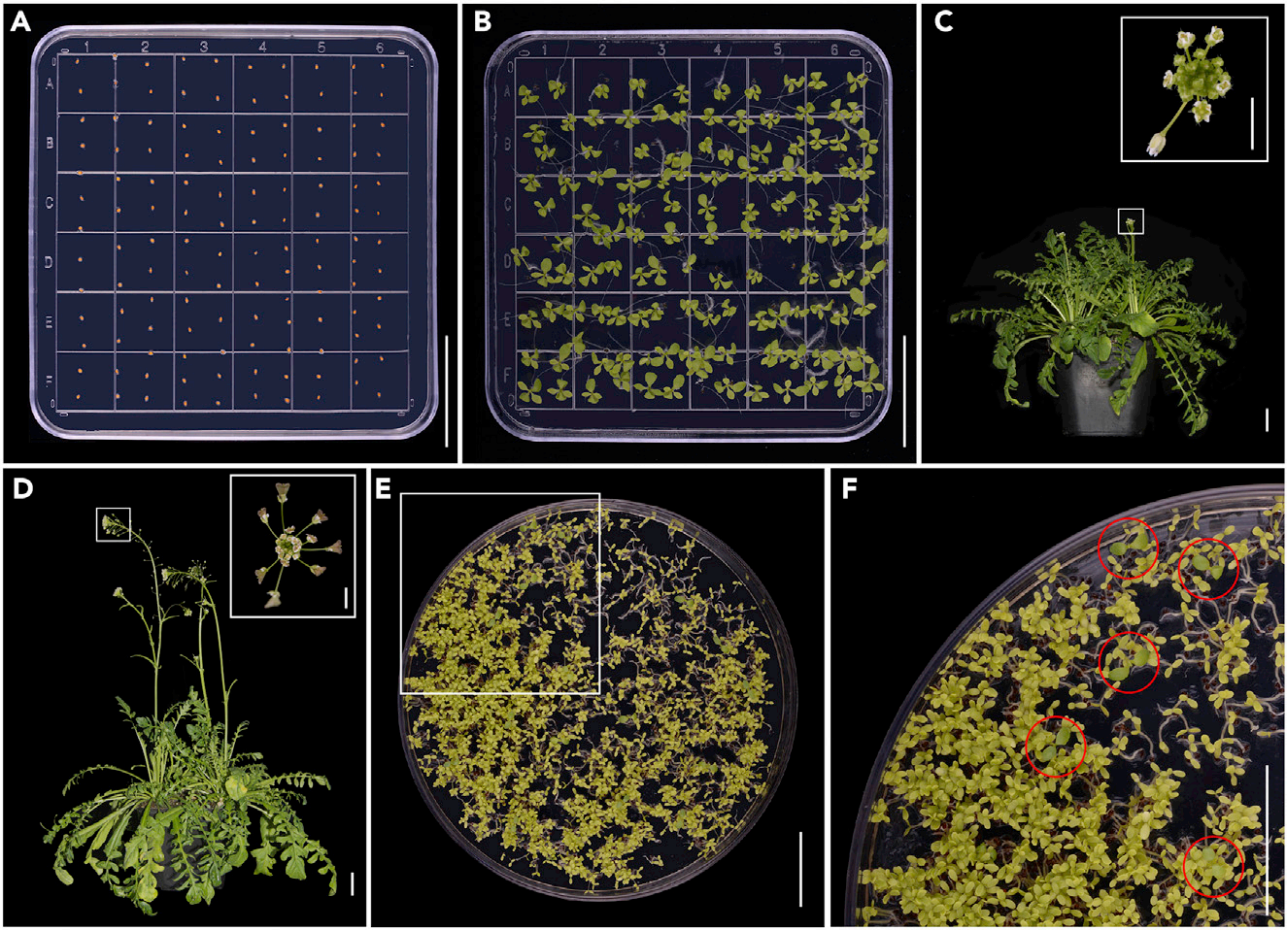
Critical steps in the *Capsella* transformation process (A) The sterilized seeds were planted evenly on the MS agar plate. (B) The ～10-day-old seedlings on the MS agar plate showing the stage ready for transplanting. (C) The plants on the bolting stage that were subject the first dipping, the insertion shows an enlarged inflorescence for dipping. (D) The plants that were subject the second dipping, the insertion shows an enlarged inflorescence for dipping. (E and F) Screening of transformant on selection MS medium using Basta as a selection marker. (F) An enlarged picture outlined in (E) shows the green and strong positive transformants (red circles) against the yellowish negative ones. Scale bars in (A–F), 2.5 cm; insertions in (C and D), 0.5 cm.
  e. Transfer the seeds into the growth room under long-day (16 h light/8 h dark) conditions at 22°C for 12 days, the seeds are normally germinated in ∼24–48 h.
  f. The 10-day-old seedlings were transplanted to soil to grow in the growth room under long-day (16 h light/8 h dark) conditions at 22°C (Figure 2B).

13. Preparation of *Capsella* plants for floral dipping
  **Timing: ∼35–40 days**
  a. Wash the seeds once with sterilized ddH_2_O, centrifuge transiently to collect the seeds.
  b. Remove the water completely and resuspend the seeds with 5% sodium hypochlorite supplied with 0.1% Triton X-100 for 3 min, remove the sodium hypochlorite completely and wash the seeds four times with sterilized ddH_2_O.
  c. Microwave the seed growth MS medium, cool down the medium in water to ∼45°C add 20 μL 100 mM GA_3_ to a final contraction of 10 μM. Mix gently and pour the medium into the square petri dishes (10 cm) in the laminar hood.
    ***Note:*** Gibberellin (GA_3_) is required to the break the seed dormancy in *Capsella*.
    **CRITICAL:** Always add the GA_3_ when the medium cooling down to ∼45°C as GA_3_ is sensitive to high temperature.
  d. Put the sterilized seeds on MS agar plates evenly (Figure 2A), and store the seeds in the dark at 4°C for 24–48 h for stratification.
    **CRITICAL:** Stratification at 4°C for 24–48 h is required to generate unified seed germination.

14. Transformation of *Capsella*
  **Timing: ∼14–21 days**
  a. Transform the *pLhGR*>>CrIND^K124R^:3×FLAG plasmids into the *Agrobacterium tumefaciens* strain LBA4404 by cold shock method, and select the colonies on YEB medium containing 50 mg/L Kanamycin, 100 mg/L Streptomycin and 50 mg/L Rifampicin at 28°C in dark.
    ***Note:*** The LBA4404 strain grows slower in YEB medium and the activity is much higher.
    **CRITICAL:** LBA4404 strain is required for *Capsella* transformation as it increases the transformation efficiency dramatically compared with other strains, such as GV3101.
  b. Pick ∼5–6 colonies and mix them in 20 mL liquid YEB medium containing 50 mg/L Kanamycin, 100 mg/L Streptomycin and 50 mg/L Rifampicin at 28°C, 200 rpm in a growth chamber.
  c. Culture the *Agrobacterium* at 28°C for 18–24 h to 1.8–2.0 (OD_600_) and collect the bacteria by centrifugation at ∼4,500 rcf (g) for 10 min at 25°C.
  d. Resuspend the bacteria using 5% sucrose in sterilized ddH_2_O supplied with 0.03% Silwet L-77 to OD_600_ of 0.8–0.9.
    **CRITICAL:** The OD_600_ value and concentration of Silwet L-77 is key to the transformation rate in *Capsella.* Please do use the recommended value indicated in the above step.
  e. Subject *htb-1 Capsella* plants that start to bolt to the first dipping (Figure 2C), and subsequently, leave in the dark for ∼20–24 h at 22°C.
    ***Note:*** We placed the dipped plants in a black plastic bag for keeping dark and humidity (∼95%), this process is crucial for a successful transformation experiment.
  f. Repeat the floral dipping twice (Figure 2D), with at least seven days intervals between each dip.
  g. After finishing the floral dip, place the plants in the growth room under long-day (16 h light/8 h dark) conditions at 22°C until fruits and seeds are mature.
  h. Harvest and desiccate the seeds at 37°C for ∼3–5 days, then keep seeds in a dry environment at 4°C.
  i. Sterilize the seeds following the process in the aforementioned steps. step 13a and 13b.
  j. Screen the transformants on selection MS medium containing 25 mg/L DL-phosphinothricin, 200 mg/L Cefotaxime and 10 μM GA_3_. Normally, ∼5–10 positive transformants will be obtained on each plate (Figures 2E and 2F).
    ***Note:*** Cefotaxime is required to suppress the *Agrobacterium* on the seed coat, lower concentration will result in overgrowth of the *Agrobacterium*, which in turn affects the selection process.
    ***Note:*** For Hygromycin selection, 40 mg/L Hygromycin is recommended on the selection plate.
  k. The transformants should be transplanted and selfed to generate enough T_2_ plants for further experiment.

## Key resources table

**Table:** 

REAGENT or RESOURCE	SOURCE	IDENTIFIER
**Antibodies**		

Goat anti-rabbit IgG-HRP secondary antibody (1:10000)	Abcam	Cat#ab6721
Mouse monoclonal [M2] anti-FLAG-HRP antibody (1:5000)	Abcam	Cat#ab49763
Rabbit polyclonal anti-SUMO1 antibody (1:1000)	Abcam	Cat#ab5316

**Bacterial and virus strains**		

*Agrobacterium tumefaciens* strain LBA4404	N/A	N/A
DH5-alpha competent *E. coli* cells	New England Biolabs	Cat#C29871

**Experimental models: Organisms/strains**		

*Capsella rubella:* WT/htb-1, Cr22.5 background	N/A	N/A
*Capsella rubella*: *pLhGR>>*CrIND:3×FLAG (10-day-old seedlings )	This Study	N/A
*Capsella rubella: pLhGR>>*CrIND^K124R^:3×FLAG (10-day-old seedlings)	This Study	N/A

**Recombinant DNA**		

pICSL01005 (pAGM1287, L0 CDS Acceptor)	Weber et al. (2011)	Addgene: 47996
pICSL12013 (L0 Pro-6GAL4UAS)	Mark Youles	N/A
pICSL50007 (L0 3×FLAG Octapeptide)	Engler et al. (2014)	Addgene:50308
pICH44300 (L0 3′UTR+AtACT2 Terminator)	Engler et al. (2014)	Addgene:50340
pICH47751 (L1 Acceptor, Pos3)	Weber et al. (2011)	Addgene:48002
pICH11041 (L1 Module, DEX-inducible Cassette Pos1)	Laurence Tomlinson	N/A
pICH47742-Bar (L1 Module, 35S+Bar+Terminator, Pos2)	André Kuhn	N/A
pICH1766 (L1 Module, End-linker, ELE3)	Weber et al. (2011)	Addgene:48018
pICSL4723 (pAGM4723, L2 Acceptor)	Weber et al. (2011)	Addgene:48015

**Chemicals, peptides, and recombinant proteins**		

30% Acrylamide/Bis-acrylamide Solution	Sigma-Aldrich	Cat#A3699
Agar (Gelzan)	Sigma-Aldrich	Cat#G1910
Ammonium persulfate (APS)	Sigma-Aldrich	Cat#908932
*Bpi*I (BbsI)	Thermo-Fisher Scientific	Cat#ER1101
*Bsa*I-HFv2	New England Biolabs	Cat#R3733S
Carbenicillin	Solar-Bio	Cat#C8251
Cefotaxime	Coolaber	Cat#CC3251
Dexamethasone (DEX)	Sigma-Aldrich	Cat#D4902
Dithiothreitol (DTT)	Sigma-Aldrich	Cat#D9760
DL-phosphinothricin	Duchefa	Cat#P0519
DMSO	Sigma-Aldrich	Cat#D8418
EDTA	Sigma-Aldrich	Cat#E5134
Ethanol	Sigma-Aldrich	Cat#459836
Ethyl methanesulphonate (EMS)	Sigma-Aldrich	Cat#M0880
Formaldehyde	Sigma-Aldrich	Cat#F8775
Gibberellic acid (GA_3_)	Sigma-Aldrich	Cat#G7645
Glycine	Sigma-Aldrich	Cat#G8898
IGEPAL (NP-40)	Sigma-Aldrich	Cat#I8896
Kanamycin	Sigma-Aldrich	Cat#60615
KCl	Sigma-Aldrich	Cat#V900068
KH_2_PO_4_	Sigma-Aldrich	Cat#P5655
Methanol	Sigma-Aldrich	Cat#322415
Na_2_HPO_4_	Sigma-Aldrich	Cat#V900061
NaCl	Sigma-Aldrich	Cat#S5886
NaF	Merck Millipore	Cat#01-0372-00
NaOH	Sigma-Aldrich	Cat#901915
N-Ethylmaleimide	Sigma-Aldrich	Cat#04259
Phenylmethylsulfonyl fluoride (PMSF)	Roche	Cat#10837091001
Phusion High-Fidelity DNA polymerase	New England Biolabs	Cat#M0530L
Protease Inhibitor Cocktail	Roche	Cat#11836170001
Silwet L-77	Coolaber	Cat#CS9791
Sodium dodecyl sulfate (SDS)	Sigma-Aldrich	Cat#L3771
Sodium hypochlorite	Macklin	Cat#MK-S828471
Spectinomycin	Sigma-Aldrich	Cat#S0692
Streptomycin	Sigma-Aldrich	Cat#S6501
Sucrose	Sigma-Aldrich	Cat#V900116
T4 Ligase	New England Biolabs	Cat#M0202L
TEMED	Sigma-Aldrich	Cat#T9281
Tris-base	Merck Millipore	Cat#648310
Triton X-100	Sigma-Aldrich	Cat#T8787
Tween-20	Sigma-Aldrich	Cat#P1379
Blotting-Grade Blocker (Non-fat milk)	BIO-RAD	Cat#706404
IPTG solution (50 mg/mL)	Coolaber	Cat#SL3860
LB liquid Medium	Coolaber	Cat#PM0010L
Murashige & Skoog (MS) Basal Medium	Coolaber	Cat#PM1011
Pierce ECL Western Blotting Substrate	Thermo Fisher Scientific	Cat#32209
Rifampicin solution (50 mg/mL)	Coolaber	Cat#SL3881
X-gal solution (20 mg/mL)	Biosharp	Cat#BL546A
YEB liquid Medium	Bioroyee	Cat#CM0395

**Oligonucleotides**		

CrIND-GG-F	Sigma-Aldrich	AATGAAGACATAATGGAGCCTCAACCTCATA
CrIND-mu-F	Sigma-Aldrich	TGAAAACTGGGCTCTAGAACATGC
CrIND-mu-R	Sigma-Aldrich	CATGTTCTAGAGCCCAGTTTTCAC
CrIND-GG-R	Sigma-Aldrich	AATGAAGACATCGAAGTTTGGGAGTTGTGGTAA

**Critical commercial assays**		

QIAGEN Plasmid Midi Kit	QIAGEN	Cat#12143
QIAquick Gel Extraction Kit	QIAGEN	Cat#28704

**Other**		

15 mL conical tube	Corning	Cat#430791
Anti-FLAG [M2] Magnetic Beads	Sigma-Aldrich	Cat#M8823
0.22 μm filter	Merck Millipore	Cat#SLGPR33RB
Magnetic rack	GE Healthcare	Cat#28-9489-64
Miracloth	Merck Millipore	Cat#475855
PVDF membrane	GE Healthcare	Cat#10600021
X-ray film	Kodak	Cat#4741019289
Low-temperature centrifugate	eppendorf	Cat#5427 R
Large space fridge	Haier	Cat#HYC-390R
3D gyratory rocker	MIULAB	Cat#RH-18
Orbital shaker	HYCX	Cat#CS-100
Protein electrophoresis system	BIO-RAD	Cat#16580
Vacuum concentrator (minimum to 100 mbar)	ScanVac	ScanSpeed32
Thermostatic bath (25°C–105°C)	So	Cat#TMSY-1
Chemiluminescence reaction system	PROTEC	OPTIMAX

## Materials and equipment

**Essential equipment:** Low-temperature centrifugate, large space fridge or cold room, 3D gyratory rocker, orbital shaker, protein electrophoresis system, vacuum concentrator (minimum to 100 mbar), magnetic rack and thermostatic bath (25°C–105°C).

### Reagent setup

**Timing: ∼1–2 days**

• Antibiotics preparation
  ○ Prepare Kanamycin solution

Dissolve 100 mg Kanamycin powder in 2 mL ddH_2_O. Sterilize using 0.22 μm filters in a laminar hood to a final concentration of 50 mg/mL. Aliquot (500 μL for each) and store at −20°C up to six months.

○ Prepare Carbenicillin solution

Dissolve 200 mg Carbenicillin powder in 2 mL ddH_2_O to a final concentration of 100 mg/mL. Sterilize using 0.22 μm filters in a laminar hood. Aliquot (500 μL for each) and store at −20°C up to six months.

○ Prepare Spectinomycin solution

Dissolve 200 mg Spectinomycin powder in 2 mL ddH_2_O to a final concentration of 100 mg/mL. Sterilize using 0.22 μm filters in a laminar hood. Aliquot (500 μL for each) and store at −20°C up to six months.

○ Prepare Streptomycin solution

Dissolve 200 mg Streptomycin powder in 2 mL ddH_2_O to a final concentration of 100 mg/mL. Sterilize using 0.22 μm filters in a laminar hood. Aliquot (500 μL for each) and store at −20°C.

○ Prepare Cefotaxime solution

Dissolve 1.0 g Cefotaxime powder in 5 mL ddH_2_O to a final concentration of 200 mg/mL. Sterilize using 0.22 μm filters in a laminar hood. Aliquot (500 μL for each) and store at −20°C up to six months.

• Plant growth related reagents
  ○ Prepare seedling growth MS medium

Dissolve 4.4 g Murashige & Skoog Basal Medium (with vitamins), 10 g sucrose in 800 mL ddH_2_O, adjust pH to 5.8 with 1 M NaOH. Add 4 g agar (gelzan), fill up to 1 L with ddH_2_O, aliquot (200 mL each) in 250 mL flask and autoclave at 121°C for 20 min. Store at room temperature (25°C) up to 8 weeks.

**Table:** 

Reagent	Final concentration	Amount
Murashige & Skoog Basal Medium (with vitamins)	4.4 g/L	4.4 g
sucrose	1%	10 g
ddH_2_O		800 mL
adjust pH to 5.8 with 1 M NaOH		
agar (gelzan)	4 g/L	4 g
ddH_2_O		up to 1 L

○ Prepare the MS liquid medium

Dissolve 4.4 g Murashige & Skoog Basal Medium (with vitamins), 10 g sucrose in 800 mL ddH_2_O, adjust pH to 5.8 with 1 M NaOH, fill up to 1 L with ddH_2_O, aliquot (100 mL each) in 250 mL flask and autoclave at 121°C for 20 min. Store at room temperature (25°C) up to eight weeks.

**Table:** 

Reagent	Final concentration	Amount
Murashige & Skoog Basal Medium (with vitamins)	4.4 g/L	4.4 g
sucrose	1%	10 g
ddH_2_O		800 mL
adjust pH to 5.8 with 1 M NaOH		
ddH_2_O		up to 1 L

○ Prepare 100 mM Gibberellic acid (Solution)

Dissolve 34.6 mg Gibberellic acid (GA_3_) in 1 mL ethanol in a laminar hood. Store at −20°C up to six months.

○ Prepare 10 mM Dexamethasone (DEX)

Dissolve 4 mg DEX in 1.0 mL ethanol in a laminar hood. Store at −20°C up to two months.

○ Prepare 20% Triton X-100

Dilute 1 mL 100% Triton X-100 with 4 mL sterilized ddH_2_O to 5 mL 20% Triton X-100. Store at room temperature (25°C) up to six months.

• Sample fixation related reagents
  ○ Prepare 10× PBS

For preparation of 10× PBS, dissolve 75.9 g NaCl, 25.1 g Na_2_HPO_4_·12H_2_O and 4.7 g NaH_2_PO_4_·2H_2_O in 800 mL ddH_2_O, adjust pH to 7.4 with NaOH, and fill up to 1 L with ddH_2_O. Sterilize by autoclaving for 20 min at 121°C. Store at room temperature (25°C) up to six months.

**Table:** 

Reagent	Final concentration	Amount
NaCl	1.3 M	75.9 g
Na_2_HPO_4_·12H_2_O	70 mM	25.1 g
NaH_2_PO_4_·2H_2_O	30 mM	4.7 g
ddH_2_O		800 mL
adjust pH to 7.4 with NaOH		
ddH_2_O		up to 1 L

○ Prepare fixing buffer in 10× PBS

Make the fixing buffer right before use.

**Table:** 

Reagent	Final concentration	Amount
10× PBS	1×	10 mL
Formaldehyde	1%	2.65 mL
ddH_2_O		up to 100 mL

○ Prepare 2 M Glycine

Dissolve 15.1 g Glycine in 70 mL ddH_2_O and add up to 100 mL. Sterilize by autoclaving for 20 min at 121°C. Store at 4°C up to six months.

• Protein extraction and immunoprecipitation related reagents
  ○ Prepare 1 M Tris-HCl pH 7.4

Dissolve 121.0 g Tris in 800 mL ddH_2_O, adjust pH to 7.4 with HCl, and fill up to 1 L with ddH_2_O. Autoclave at 121°C for 20 min. Store at room temperature (25°C) up to six months.

○ Prepare 0.5 M Ethylenediaminetetraacetic acid (EDTA) pH 7.4

Dissolve 146.1 g EDTA in 800 mL ddH_2_O, and fill up to 1 L with ddH_2_O. Autoclave at 121°C for 20 min. Store at room temperature (25°C) up to six months.

***Note:*** EDTA only dissolves in water in an alkaline environment, add NaOH pellets to pH∼=9 then adjust the pH down to 7.4 with HCl.

○ Prepare 2.5 M NaCl

Dissolve 146.1 g NaCl in 800 mL ddH_2_O and fill up to 1 L with ddH_2_O. Autoclave at 121°C for 20 min. Store at room temperature (25°C) up to six months.

○ Prepare 20% IGEPAL CA-630 (NP-40)

Dilute 1 mL 100% NP-40 with 4 mL sterilized ddH_2_O to 5 mL 20% NP-40. Store at room temperature (25°C) up to six months.

○ Prepare 0.5 M NaF

Dissolve 21.0 g NaF in 900 mL ddH_2_O and fill up to 1 L with ddH_2_O. Autoclave at 121°C for 20 min. Store at room temperature (25°C) up to six months.

○ Prepare 1 M Dithiothreitol (DTT)

Dissolve 1.543 g DTT in 10 mL of sterilized ddH_2_O. Make 1.0 mL aliquots and store at −20°C up to six months.

○ Prepare 100 mM Phenylmethylsulfonyl fluoride (PMSF)

Dissolve 0.174 g PMSF in 10 mL dimethyl sulfoxide (DMSO). Make 1.0 mL aliquots and store at −20°C up to six months.

***Note:*** Due to toxicity of PMSF and DMSO, the PMSF solution should be prepared in a fume hood.

○ Prepare 100× Complete Protease Inhibitor Cocktail

Dissolve 1 tablet in 500 μL sterilized ddH_2_O. Store at −20°C up to two months.

○ Prepare 2 M N-Ethylmaleimide (NEM)

Dissolve 0.25 g NEM in 1.0 mL ethanol. Store at −20°C up to two months.

***Note:*** NEM is toxic and an irritant, wear PPEs and conduct this step in a fume hood.

○ Prepare GTEN buffer

For 50 mL GTEN buffer, mix 5 mL 100% Glycerol, 1.25 mL 1 M Tris-HCl (pH=7.4), 100 μL 0.5 M EDTA (pH=7.4), 3 mL 2.5 M NaCl, 375 μL 20% NP-40, 100 μL 0.5 M NaF, and add 40.3 mL sterilized ddH_2_O. Store at 4°C up to two months.

**Table:** 

Reagent	Final concentration	Amount
100% Glycerol	10%	5 mL
1 M Tris-HCl (pH=7.4)	25 mM	1.25 mL
0.5 M EDTA (pH=7.4)	1 mM	100 μL
2.5 M NaCl	150 mM	3 mL
20% NP-40	1.5%	375 μL
0.5 M NaF	1 mM	100 μL
ddH_2_O		40.3 mL

○ Prepare protein extraction buffer

For 10 mL protein extraction buffer, mix 100 μL 1 M DTT, 100 μL 100 mM PMSF, 100 μL 100× Complete Protease Inhibitor Cocktail, 100 μL 2 M NEM, and fill up to 10 mL with GTEN buffer.

**Table:** 

Reagent	Final concentration	Amount
1 M DTT	10 mM	100 μL
100 mM PMSF	1 mM	100 μL
100× Complete Protease Inhibitor Cocktail	1×	100 μL
2 M NEM	20 mM	100 μL
GTEN buffer		up to 10 mL

***Note:*** The protein extraction buffer should be fresh-prepared and pre-cooled on ice before use.

**CRITICAL:** NEM is required in the protein extraction buffer as it dramatically inhibit SUMO protease activity in the cell lysate.

○ Prepare 10 mL IP wash buffer

For 10 mL IP wash buffer, mixed 1 μL 1 M DTT, 100 μL 100 mM PMSF, 100 μL 100× Protease Inhibitor, and fill up to 10 mL with GTEN buffer. Store at 4°C for 12 h.

**Table:** 

Reagent	Final concentration	Amount
1 M DTT	0.1 mM	1 μL
100 mM PMSF	1 mM	100 μL
100× Complete Protease Inhibitor	1×	100 μL
GTEN buffer		up to 10 mL

• Western-blot related reagents
  ○ Prepare 1.5 M Tris-HCl pH 8.8

Dissolve 181.5 g Tris in 800 mL ddH_2_O, adjust pH to 8.8 with HCl, and fill up to 1 L with ddH_2_O. Autoclave at 121°C for 20 min. Store at room temperature (25°C) up to six months.

○ Prepare 1.0 M Tris-HCl pH 6.8

Dissolve 121.0 g Tris in 800 mL ddH_2_O, adjust pH to 6.8 with HCl, and fill up to 1 L with ddH_2_O. Autoclave at 121°C for 20 min. Store at room temperature (25°C) up to six months.

○ Prepare 10% protein separation gel

For making 10 mL 10% separation gel, mix in order as written 4.0 mL ddH_2_O, 2.5 mL 1.5 M Tris-HCl (pH8.8), 3.3 mL 30% Acrylamide/Bis-acrylamide Solution, 100 μL 10% SDS, 100 μL 10% APS and 4 μL TEMED, mix gently.

**Table:** 

Reagent	Final concentration	Amount
1.5 M Tris-HCl (pH8.8)	375 mM	2.5 mL
30% Acrylamide/Bis-acrylamide Solution	10%	3.3 mL
10% SDS	0.1%	100 μL
10% APS	0.1%	100 μL
TEMED		4 μL
ddH_2_O		4 mL

***Note:*** The 10% protein separation gel should be fresh-prepared.

○ Prepare 5% protein stacking gel

For making 4 mL 5% protein stacking gel, mix in the following order: 2.75 mL ddH_2_O, 500 μL 1 M Tris-HCl (pH6.8), 670 μL 30% Acrylamide/Bis-acrylamide Solution, 40 μL 10% SDS, 40 μL 10% APS and 4 μL TEMED, mix gently.

**Table:** 

Reagent	Final concentration	Amount
1 M Tris-HCl (pH6.8)	100 mM	500 μL
30% Acrylamide/Bis-acrylamide Solution	5%	670 μL
10% SDS	10%	40 μL
10% APS	10%	40 μL
TEMED		4 μL
ddH_2_O		2.75 mL

***Note:*** The 5% protein stacking gel should be fresh-prepared.

○ Prepare 10× SDS running buffer

For preparation of 10× SDS running buffer, dissolve 30.3 g Tris-base, 144.0 g Glycine and 10.0 g SDS with 1 L ddH_2_O. Sterilize by autoclaving for 20 min at 121°C. Store at room temperature (25°C) up to two months.

**Table:** 

Reagent	Final concentration	Amount
Tris-base	250 mM	30.3 g
Glycine	1.92 M	144.0 g
SDS	35 mM	10.0 g
ddH_2_O		1 L

○ Prepare 1× SDS running buffer

Dilute 10× SDS running buffer to 1× SDS running buffer with sterilized ddH_2_O.

***Note:*** The 1× running buffer should be prepared fresh immediately before use.

○ Prepare 10× transfer buffer

For making 10× transfer buffer, dissolve 30.3 g Tris-base and 144.0 g Glycine with water up to 1 L. Sterilize by autoclaving for 20 min at 121°C. Store at room temperature (25°C) up to two months.

**Table:** 

Reagent	Final concentration	Amount
Tris-base	250 mM	30.3 g
Glycine	1.92 M	144.0 g
ddH_2_O		1 L

○ Prepare 1× transfer buffer

Dilute 10× transfer buffer with 200 mL methanol and 700 mL ddH_2_O to make 1 L 1× transfer buffer. Store at room temperature (25°C) up to six months.

***Note:*** The 1× transfer buffer should be pre-cooled at 4°C before use.

○ Prepare 20× TBS buffer

For preparation of 20× TBS buffer, mix 160.1 g NaCl, 4.0 g KCl and 500 mL 1 M Tris-HCl (pH8.0), and add ddH_2_O to 1 L. Sterilize by autoclaving for 20 min at 121°C. Store at room temperature (25°C) up to two months.

**Table:** 

Reagent	Final concentration	Amount
NaCl	2.74 M	160.1 g
KCl	54 mM	4.0 g
1 M Tris-HCl (pH8.0)	0.5 M	500 mL
ddH_2_O		up to 1 L

○ Prepare 1× TBST buffer

Dilute 20× TBS buffer to 1× TBS buffer with sterilized ddH_2_O and add 1 mL of Tween-20 to a final concentration of 1%.

○ Prepare 5% non-fat milk blocking buffer

Dissolve 2.5 g Blotting-Grade Blocker in 50 mL 1× TBST buffer, mix gently and store at 4°C up to 48 h.

## Step-by-step method details

### SUMOylation test: Prepare seedlings and DEX treatment

**Timing: ∼12–13 days**

In the following section, we will describe on how to prepare the plant material for the protein extraction.

1. Sterilize the T2 seeds of *pLhGR*>>CrIND^K124R^:3×FLAG using the aforementioned methods.
2. Screen the seeds on selection MS medium containing 25 mg/L DL-phosphinothricin, 200 mg/L Cefotaxime and 10 μM GA_3_.
3. Put the seeds evenly on the medium and store the seeds in dark at 4°C for 24–48 h for stratification.
4. Transfer the seeds into the growth room under long-day (16 h light/8 h dark) conditions at 22°C for 12 days, the seeds are normally germinated in ∼24–48 h (Figure 3A).

**Figure 3:**
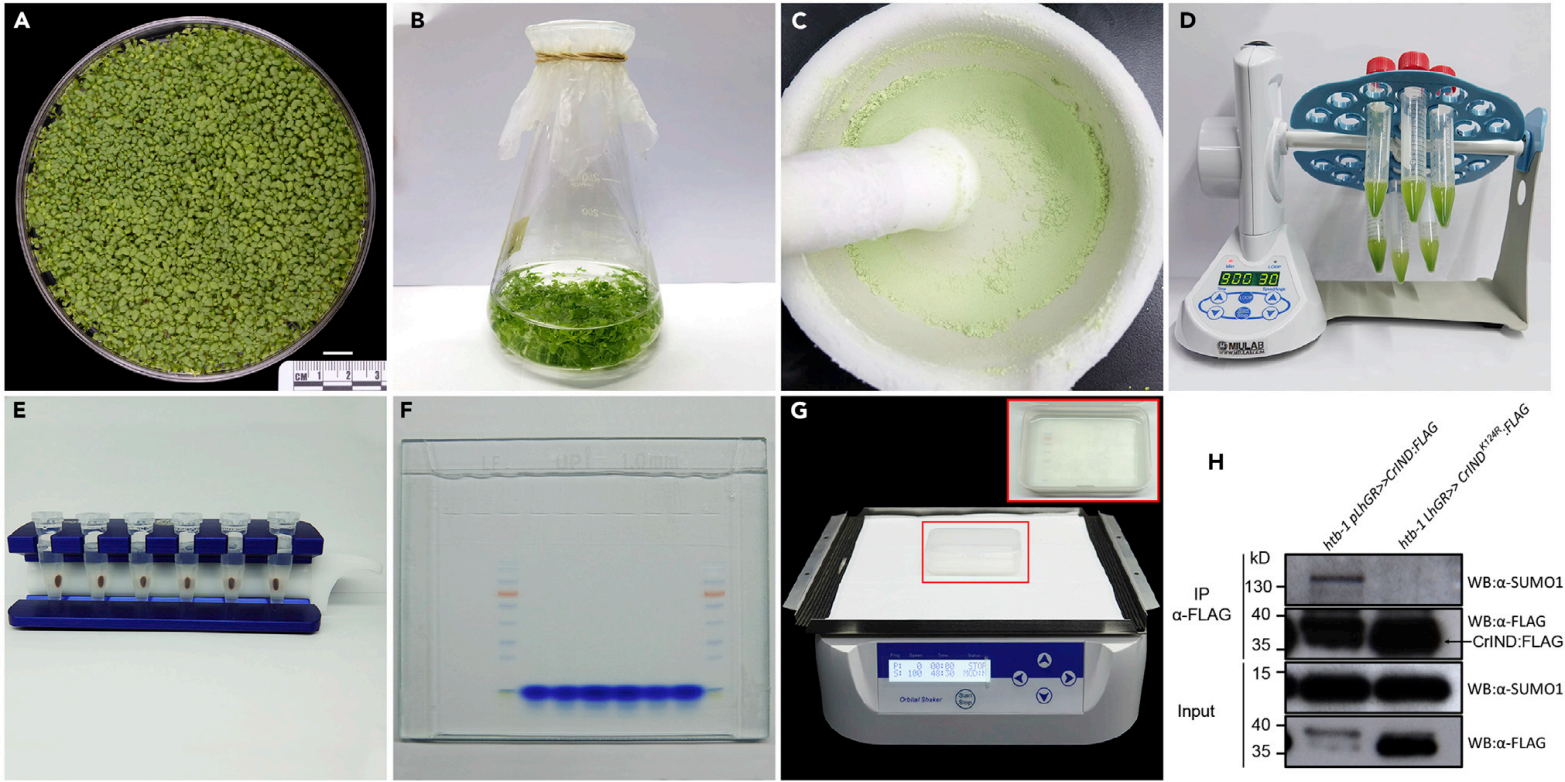
Detection of protein SUMOylation using the two component DEX-inducible system (A) ～10-day-old seedlings of the *pLhGR*>>CrIND^K124R^:3×FLAG transgenic lines ready for DEX-treatment. (B) The seedlings were collected into the flask and suspended in MS liquid medium plus 10 μM DEX. (C) The DEX-treated seedlings were fixed and ground into fine powder. (D) Protein extraction process on the rotator. (E) Collecting the anti-FLAG M2 magnetic beads on the magnetic rack after immunoprecipitation. (F) An example of SDS-PAGE gel after electrophoresis. (G) Blocking the PVDF membrane in blocking buffer (5% non-fat milk) on the shaker, the red box shows the enlarged picture of the membrane. (H) SUMOylation status test of CrIND proteins. FLAG-tagged proteins were immunoprecipitated using anti-FLAG beads. Immunoblots were probed with anti-FLAG or anti-SUMO1 antibodies. The SUMOylation of the protein were seen as a protein band with higher molecular weight using anti-SUMO1 antibodies and such a band was diminished by K124R mutation. Scale bar in (A) represents 1 cm.
5. Put 100 μL 10 mM DEX into 100 ml DEX treatment MS liquid medium. Set a mock experiment by putting 100 μL 100% ethanol into the DEX treatment MS medium.
6. Collect the 10-day-old seedlings (∼500 individuals per treatment) and put them into the DEX MS liquid medium and DEX mock MS liquid medium, respectively (Figure 3B). Fix the flasks on the shaker, grow the seedlings at 80 rpm in the growth room under long-day (16 h light/8 h dark) conditions at 22°C for ∼12–16 h.

### SUMOylation test: Total protein extraction from seedlings

**Timing: ∼2.5 h**

In the following section, we will describe the details on how to extract the total proteins from the DEX-induced seedlings.

7. Dilute 10× PBS buffer to 1× PBS buffer with sterilized ddH_2_O and add 37% formaldehyde to a final concentration of 1% to make the 100 mL fixative buffer.

***Note:*** Fixative must be freshly prepared. Seal the fixative after the preparation since formaldehyde is toxic and evaporative.

8. Harvest the DEX-treated seedlings, wrap the sample in a single layer of miracloth and cross-link the proteins in the fixing buffer using 50 mL beaker under vacuum (minimum to 100 mbar) for 15 min. Release the vacuum slowly to avoid any damage to plant tissues.

***Note:*** As the SUMOylation is a reversible and highly dynamic process in the cell, we recommend fix the proteins using 1% formaldehyde under vacuum for 15 min.

9. Stop the fixing process by adding 6.25 mL 2 M Glycine to a final concentration of 125 mM under vacuum for another 5 min.
10. Rinse the sample 3 times with ddH_2_O, dry the sample with lab tissues and weigh the sample.

***Note:*** As paraformaldehyde is toxic and evaporative, wear a mask and change the buffers in the fume hood.

11. Pre-cool the mortar, pestle, 15 mL falcon tubes and spatula in liquid nitrogen until they are completely cooled down.
12. Grind the sample (∼1.0 g) in liquid nitrogen ∼2–3 times using mortar and pestle into fine powder (Figure 3C).

**CRITICAL:** Grind the sample as much as you can, the finer the powder more protein will be extracted.

13. Transfer the sample powder to the cooled conical tube (15 mL) using the pre-cooled spatula.
14. Add 2.5×V (2.5 mL) protein extraction buffer into the samples.

**CRITICAL:** NEM in the protein extraction buffer is absolutely necessary for a successful SUMOylation test as it dramatically inhibits the SUMO protease activity.

15. Gently invert the tube to mix the sample powder with the protein extraction buffer.
16. Fix the tubes on the rotator and extract the protein at a speed of 40 rpm for 1 h at 4°C (Figure 3D).
17. Pre-cool the centrifuge at 4°C when extracting the proteins.
18. Centrifugate the samples at ～15,000 rcf (g) and 4°C for 10 min.
19. Collect the supernatant and split it into two 2.0 mL eppendorf tubes (∼1.5 mL/each) on ice.
20. Centrifugate the samples again at ～15,000 rcf (g) and 4°C for 5 min.
21. Transfer the supernatant to new 2.0 mL eppendorf tube on ice.

***Note:*** Repeat step 19 and 20 until the supernatant is clear.

**CRITICAL:** Always perform step 14–21 on ice as low temperature is critical to protect the proteins from degradation.

22. Take 50 μL of lysate as input and add 50 μL of 2× SDS loading buffer, boil the sample at 100°C for 5 min.

***Note:*** Open the tube lid carefully after boiling.

23. Store the input samples at −20°C.

### SUMOylation test: Immunoprecipitation with anti-FLAG M2 beads

**Timing: ∼4 h**

In this section, we will introduce the details of recombinant protein immunoprecipitation using anti-FLAG M2 magnetic beads.

24. Wash the anti-FLAG M2 magnetic beads (20 μL/g sample) ∼3 times with 1 mL IP wash buffer. For each wash, rotate the samples with a speed of 40 rpm for 5 min at 4°C.

***Note:*** Mix the anti-FLAG M2 magnetic beads gently before use. A magnetic rack is required for collecting the beads after each wash. Between each wash, please keep the samples on the magnetic rack for ∼2–3 min at 4°C to allow complete collection of the beads (Figure 3E).

25. Resuspend the anti-FLAG M2 magnetic beads with 50 μL IP wash buffer per tube. Add 50 μL beads into the lysate of each eppendorf tube, mix the samples on a rotator with a speed of 40 rpm for ∼2–3 h at 4°C.
26. Collect the anti-FLAG M2 magnetic beads with the magnetic rack. Discard the lysate and wash the beads ∼4 times using 1 mL IP washing buffer. For each wash, keep rotating the samples at a speed of 40 rpm at 4°C for 5 min.

***Note:*** Between each wash, please keep the samples on the magnetic for ∼2–3 min at 4°C to allow complete collection of the beads.

27. Add 50 μL of 2× SDS loading buffer, boil the beads at 100°C for 10 min, mix the sample from the same genotype, a total of 100 μL IP protein are collected.
28. Store the IP samples at −20°C.

### SUMOylation test: SDS-PAGE separation of proteins and membrane transfer

**Timing: ∼3.5 h**

In this section, we are going to introduce the detailed protocol on how to separate the proteins and transfer the protein onto the PVDF membrane for western-blot.

29. Put two SDS-PAGE gels (each SDS-PAGE contains a 5% stacking gel and a 10% separation gel) in the protein electrophoresis system.

***Note:*** The SDS-PAGE gels should be prepared in fresh; we don’t recommend using commercial pre-made SDS-PAGE gels for this SUMOylation test protocol.

30. Load 10 μL IP protein sample, 5 μL input sample and 2.5 μL protein marker into the SDS-PAGE gels.
31. SDS-PAGE electrophoresis at the voltage of 90 V for 1.5 h at room temperature.

***Note:*** The 1× SDS running buffer should be fresh-prepared and single use.

32. Cut the PVDF membrane and filter pater into 6.0 cm × 8.5 cm in size, rinse the membrane with 100% methanol for 2 min in a square petri dish, then gently remove the methanol and decant 1× transfer buffer (pre-cooled) in the petri dish.
33. Carefully collect the SDS-PAGE gels and put them into 1× transfer buffer (pre-cooled) in a square petri dish.
34. Soak sponge pad, filter paper in the 1× transfer buffer (pre-cooled).
35. Make the gel-membrane transfer sandwich in the clump (negative charge-sponge pad-filter paper-SDS-PAGE gel-PVDF membrane-filter paper-sponge pad-positive charge).

***Note:*** Care should be taken when putting the PVDF membrane onto the SDS-PAGE gel.

**CRITICAL:** Avoid bubbles between the SDS-PAGE gel and PVDF membrane. The bubbles could be avoided by flushing the gel with 1× transfer buffer before adding the PVDF membrane. Also, be sure that the gel and membrane are in order with the charge.

36. Transfer the proteins onto the PVDF membrane by electrophoresis at the voltage of 100 V for ∼1.5–2 h at 4°C.

**CRITICAL:** Be sure that the transfer buffer is always in cold environment. Carry out this process in the cold room or in the fridge. Also, a pre-cooled (−20°C) ice rack is recommended to put into the electrophoresis tank.

### SUMOylation test: Western blot

**Timing: ∼16 h**

In the following section, we will provide the details on how to detect the protein SUMOylation using anti-SUMO1 antibodies by western blot.

37. Collect the membrane in small WB incubation boxes and decant 10 mL 5% non-fat milk blocking buffer in each box, keep on the shaker at 4°C for 2 h (Figure 3G).

***Note:*** Check the color of the biggest protein marker on the membrane as an indication of protein transfer efficiency.

**CRITICAL:** Blocking for 2 h at 4°C is enough to reduce the unspecific backgrounds.

38. Remove the blocking buffer and add another 5 mL 5% non-fat milk blocking buffer. Dilute the antibody in the blocking buffer, 1:1,000 for anti-SUMO1 and 1:5,000 for anti-FLAG-HRP. Keep on the shaker at 4°C for ∼12–15 h.

***Note:*** anti-FLAG antibody could also be used in the step, the concentration, incubation time, temperature and secondary antibodies can be adjusted following the instructions of the anit-FLAG antibody.

**CRITICAL:** Aliquot the antibodies upon its arrival, keep the aliquots at −20°C and avoid repeated freezing and thawing.

39. Remove the primary antibody solution completely and rinse the membrane with the 1× TBST buffer three times, with 5 min each time on the shaker at 4°C.

***Note:*** The primary antibody solution can be reused in ∼3–4 times and keep in −20°C.

**CRITICAL:** Remove the primary antibody completely after each wash using a pipette, residual primary antibody on the membrane will generate high back-ground signals.

40. Decant 5 mL secondary antibody (1:10,000 anti-rabbit IgG in 5% non-fat milk blocking buffer) into the WB incubation box. Keep on the shaker at 4°C for 2 h

***Note:*** The anti-FLAG antibody has already conjugated with HRP, this step can be passed for the anti-FLAG membrane and directly go to step 39.

41. Remove the secondary antibody solution completely and rinse the membrane with the 1× TBST buffer three times, 5 min each time on the shaker at 4°C.

***Note:*** The secondary antibody solution can be reused in ∼5–6 times and keep in −20°C.

**CRITICAL:** Remove the secondary antibody completely after each wash using a pipette, residual secondary antibody on the membrane will generate high back-ground signals.

42. Add 200 μL Pierce ECL Western Blotting Substrate mixture (ThermoFisher, follow the manufactures instructions, https://assets.thermofisher.cn/TFS-Assets/LSG/manuals/MAN0011536_Pierce_ECL_West_Blot_Subs_UG.pdf) evenly on the membrane.
43. Expose the signal onto a film (Kodak) using a chemiluminescence reaction system.

***Note:*** For the anti-FLAG membrane, the explosion time normally takes ∼1 min to produce a desirable signal. For the anti-SUMO1 membrane, the input sample normally need ∼30 s to 1 min to generate the signal and the IP sample may take longer (∼15 min).

## Expected outcomes

### Generating *Capella* transformants

By using the optimized *Capsella* transformation protocol described in this paper, a number of positive transformants will be observed on the plates containing 25 mg/L DL-phosphinothricin (Basta) or 40 mg/L Hygromycin antibiotics. The overall transformation rate is around ∼0.15%, which is close to the rate described in *Arabidopsis* (Clough and Bent, 1998). In addition, the methods of plant transformation described in this paper is probably applicable to other Brassicaceae species as well.

### SUMOylation test using the two-component DEX-inducible system

The CrIND proteins will be induced significantly upon DEX treatment of the seedlings (Figure 3H). SUMOylation of the CrIND will be observed from the WB of purified proteins using anti-FLAG beads and anti-SUMO1 antibodies (Figure 3H). It should be noted that the specificity of SUMOylation has to be verified by doing a parallel experiment using a mutant protein with modified SUMOylation site (K124R) and possibly access functional relevance of the SUMOylation residual in the respective mutant background (Figure 3H).

### Advantages and potential applications

One general characteristic of SUMOylation lies in that its dynamic and reversable modification nature on the lysine residual of the target protein in that the protein size change that can be recognized by SUMO antibodies (Mukhopadhyay and Dasso, 2007). Compared with *in vitro* SUMOylation test system in *E. coli* and *in vivo* test system in tobacco leaves (Kong et al., 2017; Qu et al., 2020), which is based on the co-overexpression of the SUMOs and key components in the SUMOylation pathway, the use of DEX-inducible overexpression system provide more reliable and stable results as it using the native SUMOylation system *in planta*.

As SUMOylation process shared a lot of characteristics to that of ubiquitinoylation (Miura and Hasegawa, 2010), the experimental pipeline described herein could also be modified to test other kind of post-translational modifications, such as ubiquitinoylation and methylation, acetylation, and so on.

## Limitations

As stable transformants are required to generate enough plant material for the DEX-treatment and downstream experiment, it may take longer time (∼4–5 months) to finish this protocol than *in vitro* SUMOylation test in *E. coli* (Kong et al., 2017; Qu et al., 2020).

This protocol has the same constraints as any standard transgenic approach, in that the plant species or variety must be efficiently transformable to generate sufficient independent transformants. However, protoplast-based transient expression system could be an alternative approach for species that are recalcitrant to transformation (Zhang et al., 2011).

As SUMOylation is a fast and reversible process in the cells by the action of SUMO protease. Therefore, a SUMO protease mutant may be required before hand to generate stable results.

## Troubleshooting

### Problem 1

Few or no white clones in the L0-L2 cloning process (steps 6, 9 and 12 in “construction of two-component DEX-inducible plasmids (pLhGR)”)

### Potential solutions

Check the insertion sequence to see is there any *Bpi*I and *Bsa*I restriction site, any unexpected restriction sites will disrupt the ligation outcomes.

Always purified the PCR products from the agarose gel.

Check the plasmid extraction protocol see if the plasmids are prepared by midi-prep.

Re-calculate the relative amount (ng) of individual plasmids needed for each reaction. Each part/module has to be 2:1 ratio relative to the acceptor plasmids. For a simple calculation, please fill in your plasmid concentration in Table S2, the amount and volume will be automatically calculated.

Use higher concentration of T4 ligase and extend the reaction with more cycles (e.g., above 35 cycles) could improve the efficiency of Golden-Gate cloning.

### Problem 2

Low number or no *Capsella* transformant can be obtained (step 14 in “prepare the two-component DEX-inducible Capsella transformants”).

### Potential solutions

Always culture the *Agrobacterium* in the YEB medium.

Fertilized the plants with MS liquid medium twice (one week after transplant and one week before bolting, respectively), which will make the plant stronger and produce more seeds.

Synchronized the plant growth by chopping down the inflorescence that grow much faster.

Ensure the incubation temperature is 22°C after the dipping.

Extend the incubation time in dark to 24 h, if necessary, after the first and second dipping.

Check the concentration of DL-phosphinothricin (Basta) and Cefotaxime on the selection plate.

### Problem 3

Poor separation of the proteins on the SDS-PAGE Gel (steps 29–31 “step-by-step method details”).

Poor separation of the protein markers on the gel always indicates poor separation of the protein samples.

### Potential solutions

Wash the comb and glasses with ddH_2_O before making the stacking gels, any residual dry stacking gel left on the comb will dramatically affect the electrophoresis process.

Reset the separation and stacking gels.

Electrophorese the protein samples in the stacking gels at 80 V for ∼30 min, then change to 100 V in the separation gels.

### Problem 4

High background and low targeted signals of IP samples in the WB results (steps 30–43 in “step-by-step method details”).

### Potential solutions

Check the membrane transfer process and see if there are any bubbles between the gel and the PVDF membrane.

Blocking the membrane at room time temperature for 4 h.

Completely remove the primary and secondary antibodies, and wash the membrane 4 times after the antibody incubation.

Increase the amount of IP samples (up to 20 μL) loading in the gel.

Increase the amount of samples used for immunoprecipitation and use 5× SDS loading buffer to elute the proteins.

It is possible that the concentration of antibodies is too low or the duration of antibody incubation is not adequate. The concentration and incubation time of antibodies should be adjusted for different proteins and plant species.

The antibodies should be aliquoted upon arrival to avoid degradation with repeated freezing and thawing.

SUMOylation is a reversable and dynamic post-translational modification controlled by SUMO proteases (Augustine and Vierstra, 2018). The SUMO protease could be suppressed by NEM, which is very evaporative in ethanol. Check the concentration of NEM added into the protein extraction buffer.

## Resource availability

### Lead contact

Further information and requests for resources and reagents should be directed to and will be fulfilled by the lead contact, Lars Østergaard (lars.ostergaard@jic.ac.uk).

### Materials availability

The *Capsella* lines and plasmids associated with this protocol are available upon request.

## Data Availability

This study did not generate unique datasets or code.

## Supplemental information

Table S1. Primers used in this study

Table S2. Golden-gate cloning reaction set-up table, related to “Construction of two-component DEX-inducible plasmids (*pLhGR*)” steps 4, 7, and 10
