# Supplementary material for: An optimized protocol to assess SUMOylation in the plant Capsella rubella using two-component DEX-inducible transformants
Source: STAR Protoc. 2022 Feb 26;3(1):101197. doi: 10.1016/j.xpro.2022.101197 (PMC8885766; doi:10.1016/j.xpro.2022.101197)
Supplement: Table S1. Primers used in this study [file mmc1.pdf]

Supplemental Table S1. Primers used in this study, related to "Construction of two-component DEX-inducible plasmids (*pLhGR*)" step 1.

Primer Name	Sequences	Introduction
CrIND-FLAG-GG-F	AATGAAGACATAATGATGGAGCCTCAACCTCATAA	Golden-gate L0 reaction
CrIND-FLAG-GG-R	AATGAAGACATCGAACAGGTTTGGGAGTTGTGGTA	Golden-gate L0 reaction
L0-SeqF	CGTTATCCCCTGATTCTGTGGATAAC	L0 plasmid sequencing
L0-SeqR	GTCTCATGAGCGGATACATATTTGAATG	L0 plasmid sequencing
L1-SeqF	GAACCCTGTGGTTGGCATGCACATAC	L1 plasmid sequencing
L1-SeqR	CTGGTGGCAGGATATATTGTGGTG	L1 plasmid sequencing
L2-SeqF	GTGGTGTAAACAAATTGACGC	L2 plasmid sequencing
L2-SeqR	GGATAAACCTTTTCACGCCC	L2 plasmid sequencing

Cloning of CDS of the gene of interest
L0 acceptor pICSL01005-Spe (AATG-TTCG)
***Bpi*I** for L0 reaction:
5'-GAAGAC-N2^-3'
3'-CTTCTG-N6^-5'
The gray shaded base pair are the protection bases

Note: The pICSL01005 plasmids ending TTCG overhangs
The last three bases (TCG) of the overhang will encode a Ser residue. The T in the first position of the TTCG overhang will therefore be the third position of the last codon of the part. To make your protein in-frame with the tag, you could:
a. **DELETE THE STOP CODON** and **INCLUDE TWO ADDITIONAL BASE-PAIRS** to make a new codon of which the last position is the first T from the TTCG overhang, thus making a two-codon linker.
b. **DELETE THE STOP CODON** and **REMOVE THE LAST BASE PAIR** of the last codon before the stop codon and allow it to be replaced with the first T from the TTCG overhang. Pay attention to what amino acid this codon will make as introducing a structural/charged base may interfere with folding or function.

It should be noted that both a and b could be applied in designing the primers, and we used method b in the present study.
